# Genetic architecture of dispersal behaviour in the post-harvest pest and model organism *Tribolium castaneum*

**DOI:** 10.1038/s41437-023-00641-6

**Published:** 2023-07-29

**Authors:** Michael D. Pointer, Lewis G. Spurgin, Matthew J. G. Gage, Mark McMullan, David S. Richardson

**Affiliations:** 1 University of East Anglia, Norwich, UK; 2 Earlham Institute, Norwich, UK

**Keywords:** Experimental evolution, Evolutionary ecology

## Abstract

Dispersal behaviour is an important aspect of the life-history of animals. However, the genetic architecture of dispersal-related traits is often obscure or unknown, even in well studied species. *Tribolium castaneum* is a globally significant post-harvest pest and established model organism, yet studies of its dispersal have shown ambiguous results and the genetic basis of this behaviour remains unresolved. We combine experimental evolution and agent-based modelling to investigate the number of loci underlying dispersal in *T. castaneum*, and whether the trait is sex-linked. Our findings demonstrate rapid evolution of dispersal behaviour under selection. We find no evidence of sex-biases in the dispersal behaviour of the offspring of crosses, supporting an autosomal genetic basis of the trait. Moreover, simulated data approximates experimental data under simulated scenarios where the dispersal trait is controlled by one or few loci, but not many loci. Levels of dispersal in experimentally inbred lines, compared with simulations, indicate that a single locus model is not well supported. Taken together, these lines of evidence support an oligogenic architecture underlying dispersal in *Tribolium castaneum*. These results have implications for applied pest management and for our understanding of the evolution of dispersal in the coleoptera, the world’s most species-rich order.

## Introduction

Dispersal is important in the ecology and evolution of many species (Ronce 2007) and plays a key role in species’ ability to cope with habitat fragmentation and anthropogenic climate change (Travis et al. 2013). Conversely, dispersal also drives inadvertent introductions and invasions of non-native species (Renault et al. 2018). Knowledge of the movement of pests, and its genetic basis, will also allow us to better preserve biodiversity, forecast outbreaks, and to design management and control strategies (Jeger 1999). The consequences of dispersal can be considerable for the organism; as a result of moving to different abiotic, biotic, and/or reproductive environments, individuals may experience extensive differences in fitness, but there are also effects at higher levels of organisation (i.e. at the population or species level compared to at the individual level; Clobert et al. 2012). As such, individual dispersal decisions combine to shape the distribution of individuals and populations and thus govern the area a given species occupies, defining the limits of range expansions and shifts (Kokko and López-Sepulcre 2006). Dispersal is also a key determinant of metapopulation persistence in fragmented, disturbed, or unstable environments, where it can allow recolonisation to balance local extinction (Eriksson et al. 2014). Dispersal also mediates metapopulation structure, determining migration rates and gene flow, which in turn influence evolutionary trajectories of dispersal and on-dispersal traits (Suárez et al. 2022). In both cases, the genetic architecture of the focal trait is an important factor, for example the number of controlling loci can affect the rate of dispersal evolution (Weiss-Lehman and Shaw 2022) and epistatic variance in any trait can be converted to additive variance by drift during founding events, or vice versa as a result of later additional gene flow into the population (Wade and Goodnight 1998; Hill 2017).

A genetic basis to dispersal has been shown in a wide variety of animal species, however, the identity, number, and mode of action of the genes underlying dispersal vary widely (Saastamoinen et al. 2018). Large-effect loci underlying dispersal have been identified in various species (e.g. Trefilov et al. 2000; Fidler et al. 2007; Krackow and König 2008; Edelsparre et al. 2014). Contrastingly, a powerful study of dispersal utilised the *Drosophila* Genetic Reference Panel and found 192 genes associated with variation in locomotion (Jordan et al. 2012). This finding agrees with the view that polygenic architectures generally underlie complex quantitative traits (e.g. Husby et al. 2015; Santure et al. 2015), such as dispersal. However, the genetic basis of dispersal remains obscure in most species.

*Tribolium castaneum* is a significant pest of stored food products (El-Aziz 2011), responsible for a portion of the ~10% of total grain lost to insects during storage (Boxall 2001), and therefore their control is of biological and economic importance. This species is also an important laboratory model across a range of disciplines, from evolutionary ecology to development (Denell 2008; Pointer et al. 2021). Additionally, as Coleopterans, they are members of the most species-rich order and, being an early diverging lineage in the phylogeny of metamorphosing insects, *T. castaneum* is considered highly representative of other insect species (Brown et al. 2003; Stork et al. 2015). Understanding the genetic causes and consequences of dispersal in *Tribolium* could have significant pest control benefits, as well as adding to our knowledge of how populations evolve during range expansion (Weiss-Lehman and Shaw 2022) and how insects may respond to habitat suitability shifts under climate change.

Several studies on *Tribolium* have demonstrated a genetic basis to dispersal through responses to artificial selection (Schurr and Bolduan 1967; Ogden 1970a, b; Ritte and Lavie 1977; Korona 1991; Ruckman and Blackmon 2020); though many of these measured dispersal only in single-sex groups. The rapid response to selection observed across these experiments, has led some authors to postulate that the trait is controlled by very few loci (Ogden 1970a; Ritte and Lavie 1977). For example, evidence of differential dispersal in the sexes between reciprocal crosses, have led to suggestion that dispersal is controlled by a single, sex-linked locus (Ritte and Lavie 1977). In contrast, line cross analysis on a similar set of crosses has provided evidence that epistatic interactions across loci dominate additive effects in dispersal adaptation (Ruckman and Blackmon 2020). Despite considerable effort spent investigating dispersal in *Tribolium* little is known definitively; findings are contradictory (Ritte and Lavie 1977; Ruckman and Blackmon 2020), and many studies are only modestly replicated (Ritte and Lavie 1977), and/or were conducted before the availability of modern computational and molecular genetic techniques.

Here, we combine quantitative genetic, population genetic, and computational approaches—comparing results from replicated experiments and agent-based simulations—to understand the genetic basis of *Tribolium* dispersal. Dispersal is known to consist of three distinct phases, emigration, transit, and immigration (Ronce 2007). While we acknowledge that the focus of this study and previous studies is technically emigration, local movement tendency has been linked to longer distance dispersal by flight (Zirkle et al. 1988) and we use the term dispersal for consistency with the existing *Tribolium* literature. Further, our experimental setup requires individuals to traverse an empty resource patch before they are deemed to have dispersed, making the movement trait studied here more emigration-like than previous work in the system (e.g. Ritte and Lavie 1977). Specifically, by comparing the results of artificial selection for dispersal to agent-based simulations modelling artificial selection under a range of genetic architectures we investigate the genetic basis of the trait. Next, by comparing the dispersal behaviour of experimental crosses to crosses simulated under either sex-linked or non-sex-linked architectures, we explicitly test the hypothesis of a single sex-linked dispersal locus proposed by Ritte and Lavie (1977). If this holds true we predict that simulations under a single-locus architecture will best fit the observations from experimental selection, and that behaviour of males and females will differ in the F1 generation of experimental and simulations under sex-linked architectures but not under non-sex-linked architectures. Lastly, we compare dispersal in experimentally inbred beetle lines and inbreeding simulations, gathering further evidence on the genetic basis of dispersal in this system.

## Materials and methods

### Beetles and husbandry

Beetles used were of the Krakow super-strain (KSS), bred to combine global *Tribolium castaneum* genetic variation (Laskowski et al. 2015). Stock populations were maintained at Ne ≈ 300 within 1.2 L plastic containers covered with lids into which 70 × 70 mm windows of fine mesh had been inserted for ventilation. Populations were kept on a ‘standard fodder’ medium of 90% organic wheat flour and 10% brewer’s yeast at 30°C, 60% relative humidity, and a 12:12 light-dark cycle (light from 8 am–8 pm) at the University of East Anglia. These are the same conditions under which the stock populations have been kept for ~12 years. The standard husbandry cycle consisted of two phases; during the oviposition phase adult (12+/−3 days post-eclosion) beetles chosen to parent the next generation were removed from their populations and placed into fresh fodder for seven days of mating and egg-laying. Following this period, adults were sieved from the fodder and discarded, beginning the 35-day development phase**—**during which time eggs in the fodder developed through the larval and pupal stages to become adults. By preventing any interaction between sexually mature adults and offspring reduces the risk of negative density-dependent effects, removes the opportunity for intergenerational interactions, such as egg-cannibalism, and allows accurate tracking of passing generations.

### Experimental methods**—**dispersal phenotyping assay

**Fig. 1:**
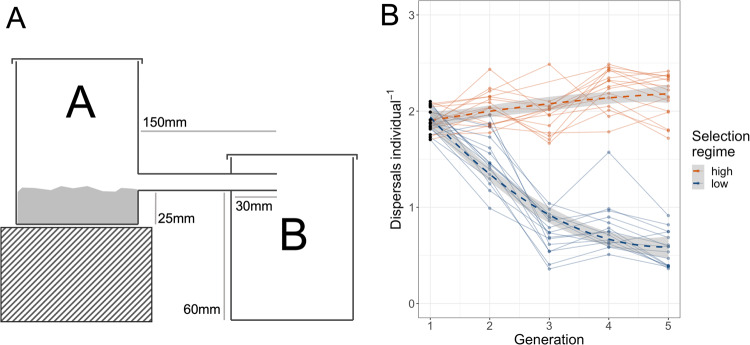
Artificial selection on dispersal. **A** Experimental arena setup used to assay the dispersal behaviour of experimental *Tribolium castaneum* populations and provide a basis on which to artificially select individuals displaying high and low dispersal propensity. **B** Mean dispersals per individual (of a maximum of three) for each *T. castaneum* selection line across four generations of selection on high and low dispersal (generation 1 represents behaviour in the stock population). Orange and blue represent high and low selection regimes respectively. Solid lines connect repeated measurements from the same selection line, while dashed lines show predictions generated by a GLM fitting the interaction of selection regime and generation modelled as a second order polynomial.

To investigate the genetic basis of dispersal behaviour and its response to selection we established selection lines by breeding from individuals based on their behavioural phenotypes, as determined using dispersal assays. Dispersal arenas were constructed that consisted of two square, 1.2 L plastic containers with removable lids, connected by a length of rigid PVC tubing with 8 mm internal diameter (Fig. 1A). When 200 ml of fodder was placed into container A and made level, the surface of the fodder intersected the opening of the tube. Stoppers made from baked Fimo polymer clay were used to block either end of the tube when required. On the first day of the assay the tube was blocked and 200 ml of fodder was placed into container A, made level and topped with oats to aid traction. Next, 200 adult beetles of mixed sex (12+/−3 days post eclosion) were added to container A and given a 2 h acclimation period. After this time, the tube was unblocked, beginning a 20 h dispersal period. The walls of both containers were smooth and prevented climbing, so individuals that fell from the tube to the floor of container B were unable to return to container A. Beetles in container B at the end of the period were considered to have dispersed. We utilised two types of dispersal assay, differing in the number of dispersal opportunities they afforded: a 1-opportunity assay, (described above) and a 3-opportunity assay, which provided greater resolution at which to measure behaviour. During the latter, following the first dispersal period, individuals from container B were marked with a dot of paint on the dorsal thorax, using a paint marker pen (Posca, 0.7 mm). The contents of container A were separated by sieving. The fodder was replaced in container A, flattened and topped with the same oats. The original 200 individuals from a single replicate, retrieved from container A or B, were returned to container A for a second round of dispersal. This process was repeated twice so each individual had three opportunities to disperse, and its number of dispersals recorded. To mitigate effects of unavoidable small differences in arena construction, lines were assigned randomly to arenas each generation. Processing each line was time-consuming so, in order to guard against time-of-day effects, lines were divided evenly between two temporal blocks (lines 1–8 for both treatments in block 1 and lines 9–16 in block 2). Some component of the measured behaviour may be due to learning across dispersal opportunities within generations, however, as any effect of learning would be consistent across generations we expect the overall impact to be minimal.

### Experimental methods**—**breeding dispersal lines by artificial selection

Initial 3-opportunity dispersal assays were conducted and the selection criteria were three dispersals for high-dispersal lines and zero dispersals for low-dispersal lines. Assays were conducted on 16 replicate groups of stock beetles, with individuals meeting the selection criteria used to found 16 high- and 16 low-dispersal lines respectively. Where possible, 30 individuals were taken to produce the next generation in each replicate of each treatment, otherwise as many as met the selection criteria. This was only ever <30 in the initial generation; of the 32 created lines, the minimum population size was 21 (mean = 27.56, SD = 3.35). In each of generations 2–5, dispersal behaviour in high- and low-dispersal lines was retested with 3-opportunity assays. From individuals meeting the selection criteria of the relevant treatment, 30 were randomly selected to produce the next generation, all other individuals were discarded.

To maximise the effectiveness of selection, it was desirable that all offspring were parented by individuals meeting the selection criteria of the relevant regime, *i.e*. from the group of 30 parental individuals. However, during the assay, individuals selected to produce the next generation could potentially mate with others not from this group, for example, females selected to propagate the high dispersal regime could have been inseminated by non-highly-dispersing males. To mitigate this, we isolated the parental group of 30 individuals for 72 h of intragroup mating, allowing the strong last male precedence displayed by *Tribolium* (Lewis and Austad 1990) to minimise the number of offspring sired by non-group males. After this time, fodder potentially containing non-group eggs was discarded and beetles were transferred to fresh fodder for 7 days of oviposition. Following oviposition, eggs were left to develop in the fodder, adults were removed, frozen, and sexed by the presence of odiferous glands on male upper forelegs (Hinton 1942). Sexing allowed us to ensure that sex ratios were not overly skewed among reproductive groups, and to evaluate sex bias in dispersal behaviour itself. Using this procedure 16 high-dispersal and 16 low-dispersal lines were selected over 5 generations. Hereafter, referred to as 1–16H and 1–16 L respectively.

After generation 5, a 1-opportunity assay was used every other generation (to gen 15) to monitor dispersal propensity and select 30 individuals to produce the next generation. In non-assayed generations, 100 individuals were randomly selected to propagate each line. Selection lines were housed within 250 ml PVC containers with a circular base of 34 mm radius and 100 mm in height, round (20 mm radius) windows of fine metal mesh were inserted in the screw-on lids.

### Experimental methods**—**phenotyping crosses within and between dispersal treatments

To investigate whether dispersal was under the control of a single, sex-linked locus we performed reciprocal crosses within and between selection lines and assayed the dispersal behaviour of offspring. High and low dispersal lines were paired according to their number (1H with 1 L, *etc*.) and four types of cross (within-line and reciprocal between-line crosses; Fig. S1) were performed on each pair. We began by obtaining virgin individuals, taken as pupae from the relevant populations on day 20 of the development phase and raised to adulthood in single-sex groups in 57 ml (61 mm diameter, 27 mm height) plastic containers with hinged lids and ventilation holes. Once mature, males were marked with a coloured dot on the dorsal thorax using a paint marker (0.7 mm; Uniposca, www.posca.com), a method shown not to alter behaviour (Sales et al. 2018). At 12+/−3 days post-eclosion, a single male and female were paired in a 5 ml vial on 0.5 ml of fodder for 48 h of mating opportunity, before males were discarded and females transferred to oviposit on fresh fodder for seven days in 57 ml containers. Each type of cross between a single pair of individuals was replicated five times for each pair of lines. After the females were removed following oviposition, egg-containing fodder from the five replicate crosses was combined so that offspring development took place in non-sibling groups. Each female oviposited on 10 ml of fodder, to maintain the per-female fodder volume from the selection lines, and maintain equal developmental population density. From these new populations, pupae were sexed and kept in single-sex groups until males could be marked post-eclosion. At 8+−3 days post-eclosion 100 males and 100 females were combined to form a cohort of 200 beetles with a 1:1 sex ratio, four days later this population entered a 1-opportunity dispersal assay.

### Experimental methods**—**breeding and phenotyping inbred lines

We established highly inbred lines by repeated sib–sib mating to remove genetic variation within each line. Intense inbreeding will increase homozygosity, potentially fixing alleles at each locus. If dispersal is a single-locus trait, highly inbred lines should fix either the high or low dispersal allele, with probabilities proportional to the starting allele frequencies, and accordingly display either high or low dispersal behaviour, equivalent to that seen in artificially selected lines. By comparing the dispersal behaviour of these lines to predictions generated by simulation under different assumed genetic architectures we have another way to evaluate the genetic basis of this trait. To create the inbred lines, 110 KSS males and 110 KSS females were paired 1:1 (using the same method as for the previous experiment) and used to found 110 lines. Each generation for 10 generations, three male and three female pupae were sexed from each line at 20 days following the end of the oviposition period and left to eclose as virgins in single-sex single-line groups. Resulting adults were paired to create three replicate single-pair matings, coded A, B, and C**—**to guard against lines going extinct through failure to mate. Pupae used to parent the following generation were always taken from replicate A, unless it was extinct whereon B was used, etc. The fecundity of individual females is reduced by inbreeding depression (Fernández et al. 1995), so to obtain the 200 individuals required for a dispersal assay an additional generation was bred, from a group of 30 beetles per line. As this breeding immediately followed a single pair mating, these 30 individuals were siblings, meaning a total of 11 generations of inbreeding were conducted, following which 64 lines of the initial 110 were left extant. Dispersal behaviour of inbred lines was then measured using a 1-opportunity dispersal assay.

### Simulation methods**—**agent-based simulation to model artificial selection on dispersal

A population genetic simulation of the dispersal selection experiment was created in R (ver.2021.09.1**—**R Core Team 2020). This agent-based simulation assumed combinations of several genetic (sex-linkage state, dominance value, trait heritability, starting dispersal allele frequency) and demographic parameter values, while incorporating some stochasticity in how they affected simulated populations. By running simulations across the available parameter space we were able to compare simulated data with experimental observations and make qualitative estimates of unknown parameters within our experimental populations (for details see supplementary material).

To estimate the contribution of dominance, heritability, and starting allele frequency to dispersal outcomes, we used the simulation model to perform a parameter scan across a range of values, so that we might compare the response to selection of dispersal behaviour in simulated populations to that observed in experimental populations. Parameter scans comprised a model run for each of 100 different combinations of dominance, heritability, and starting dispersal allele frequency values (Table S1). Scenarios containing allele frequencies of zero and one, or a heritability of zero, were excluded because evolution is not possible under these conditions.

To investigate the number of genes that might underlie dispersal, we developed versions of the simulation model that each assumed a different trait architecture, where dispersal was controlled by 1, 3, 5, and 10 additive, unlinked, biallelic loci, each inherited as described above, with no recombination. For architectures with multiple loci, an individual’s dispersal probability was the mean of probabilities calculated independently for each locus. Parameter scans were carried out for each architecture, where each consisted of 50 independent repeats (each equivalent to one high and one low line from the experimental selection) and simulated populations were tracked across 4 generations of selection, to mirror the experimental procedure. For the single locus model, we studied an additional case where the trait was X-linked. *Tribolium castaneum* follows the XX/XY sex-determination system (Juan and Petitpierre 1991). Here females received two alleles, which combined to determine their phenotype, as described above, but males received only one dispersal-determining allele and a Y, which did not contribute to the dispersal phenotype.

### Simulation methods**—**phenotyping crosses within and between dispersal treatments

The simulation model was extended to mirror the crosses performed with experimental beetle lines. This allowed us to generate predictions of the dispersal behaviour of offspring for comparison with results from experimental crosses. For each of the sex-linked and non-sex-linked versions of the single-locus architecture, we began by taking the parameter scan outputs from scenarios that best approximated the experimental data**—**for both architectures this was *h* = 0.6, *d* = 0.5, *A* = 0.8 (see results). These outputs contained simulated individuals that were the result of four generations of selection. We then simulated an additional mating, in the same way as before except, where during selection females were mated to males that matched their phenotype, here females were assigned to one of four cross treatments and mated to males with corresponding phenotypes. These cross types were: within high selection regime (Hf-Hm); high selection regime female x low selection regime male (Hf-Lm); low selection regime female × high selection regime male (Hm-Lf); within low selection regime (Lf-Lm; Fig. S1). Their simulated offspring were then put through a simulated 1-opportunity dispersal assay, where binary dispersal outcomes were assigned probabilistically according to individual genotype, dominance and heritability.

### Simulation methods**—**simulating and phenotyping inbred lines

This simulation was adapted from the dispersal selection simulation, and simulated 10 generations of inbreeding through individual sibling-sibling matings and one additional generation parented by 30 individuals. Following inbreeding, a 1-opportunity dispersal assay was simulated on inbred lines, in the same way as described for the dispersal selection model. This was done separately for 250 replicates of each trait architecture (single-, 3-, 5- and 10-locus), using all sets of parameters values for each that gave highest agreement with experimental results during the parameter scan (*R*^2^ > 0.8). In this way we generated predictions about the dispersal behaviour of individuals of highly inbred lines, under different assumptions regarding the genetic control of the trait.

### Statistical methods

All data wrangling and analysis were performed in R ver.2021.09.1 (R Core Team 2020) and data-wrangling largely used the Tidyverse packages (Wickham et al. 2019). Generalised linear mixed models (GLMMs) were fitted using ‘Lme4’ (Bates et al. 2015) and Satterthwaite method p-values were obtained using ‘lmerTest’ (Kuznetsova et al. 2017; Luke 2017).

### Statistical methods used for experimental data

To investigate the effectiveness of selection on dispersal behaviour, we employed a GLMM. The response variable was the mean number of dispersals per beetle (maximum of 3) for each selection line each generation. Selection regime and block were entered as fixed effects, alongside generation fitted as a second-order polynomial, and the interaction of generation and selection regime. Line ID was fitted as a random factor. Additionally, a GLMM was applied separately to high-selection data to test for changes in dispersal propensity over generations. This model was fitted twice, first with generation as a linear predictor, then as a second-order polynomial.

We tested for sex bias in dispersal behaviour in the initial generation using chi-squared tests on sex ratios of 3-time and 0-time emigrants summed across all replicates. We also analysed the sex of emigrants using generalised linear models (GLMs) with a binomial error distribution and a logit link function; proportion of males was the response variable and sample sizes were entered as weightings (Wilson and Hardy 2002). The model was fitted separately to data on numbers of 0- and 3-time dispersers, with the effect of generation, fitted as a linear fixed effect, on sex bias in the high selection regime and the low selection regime.

To compare the dispersal behaviour of male and female offspring from different types of crosses between dispersal lines, we employed a GLMM with number of dispersals as the response variable. Because the density of organisms is known to affect dispersal rates (e.g. Ogden 1970b), assay-level means of disperser numbers were preferred to individual-level outcomes to avoid non-independence within replicate populations. A first model testing the individual effects of cross type and sex contained these as fixed predictors, along with block, while line ID was entered as a random predictor. A second model contained the interaction of cross type and sex, along with block as fixed predictors and line ID as a random factor. Hartigan’s dip test (Hartigan and Hartigan 1985) was used to test for multimodality in the distribution of dispersal propensity of inbred lines, with p-values obtained by 10,000 Monte Carlo simulations using R package ‘diptest’ (Maechler 2021).

### Statistical methods used for simulated data

To obtain information on the likely genetic architecture, starting allele frequency, dominance and heritability in experimental populations, we compared results from empirical selection to results of simulations. We did this by starting with the parameter estimates generated by the statistical model applied to the experimental data (above). We then fit those parameter estimates to the output of each simulated scenario to generate residuals. From these we manually computed R^2^ values, to assess how well each simulated scenario approximated the experimental data. This analysis was performed for each genetic architecture and for autosomal and sex-linked versions of the single-locus simulation. To assess whether the sex-linked architecture led to sex-biased dispersal, we computed the difference in dispersal tendency of males and females for each scenario under both single-locus simulation and compared these visually using a heatmap of parameter space. Sex difference in dispersal was computed as the mean dispersal tendency in females across replicates subtracted from the same metric computed for males.

On each output from simulated crosses between selection lines, for sex-linked and non-sex-linked single-locus architectures we employed GLMMs as for the experimental cross data (see above). However, fitting these directly resulted in singularity as the random effect of line accounted for none of the variance in the data, we therefore removed this and fitted the same fixed effects as a GLM. We also fitted the statistical model generated using the experimental dataset to the simulated output from each architecture, using predicted values to obtain residuals and calculated an *R*^2^ to compare the fits.

Hartigan’s dip tests (as above) were used to test for multimodality of distributions of (i) mean allele frequencies within lines within each genetic architecture, and (ii) of numbers of dispersers in each line. Initially, this was done using 250 replicate simulated lines, but for architectures showing significant multimodality these 250 replicates were randomly subsampled down to 64 to match the number of experimental inbred lines and retested.

## Results

### Experimental results**—**dispersal in artificially selected lines

Dispersal behaviour differed between selection regimes after only one generation of selection, as evident from the non-overlapping 95% CIs of model predictions (Fig. 1B). This was a result of dispersal falling in the low selection regime lines until generation 3, then remained low, while dispersal in the high selection lines showed a small but significant increase when generation was fitted as a linear predictor (GLMM, β = 0.07, SE = 0.01, *p* < 0.001). A significant interaction between selection regime and generation was found when the effect of generation was modelled as a quadratic (Table 1). Individual variation in dispersal propensity was present in all generations of both treatments, but a shift towards higher and lower propensities was observed in high and low lines respectively. For a comparison of results from 1- and 3-opportunity assays see supplementary material.

**Table 1:** A linear mixed model of the response to selection in experimental *T. castaneum* populations artificially selected for high and low dispersal behaviour.

	Estimate	SE	*p*
(Intercept)	2.16	0.04	<0.001
Generation	2.21	0.3	<0.001
Generation^2^	−0.75	0.3	0.013
Selection regime	−1.1	0.04	<0.001
Block	−0.06	0.04	0.146
Generation x Selection regime	−5.64	0.42	<0.001
Generation^2^ x Selection regime	6.26	0.42	<0.001

With mean dispersals per individual as the dependent variable, block was fitted as a fixed effect to account for potential variation in experimental conditions, generation was fitted as a second order polynomial and ‘high’ is the reference category for selection regime. As a random effect we modelled a random intercept of line ID (Var<0.01, SD = 0.05).

Within the low dispersal regime, we observed a male bias among dispersers (χ^2^ = 26.79, df=1, *p* < 0.001, *n* = 344; Fig. S2), which decreased across generations (β = 0.10, SE = 0.03, *p* < 0.001). The proportion of males among dispersers in high dispersal lines was lower than expected (χ^2^ = 5.14, df = 1, *p* = 0.02, *n* = 467; Fig. S2).

### Experimental results**—**phenotyping crosses within and between dispersal treatments

**Fig. 2:**
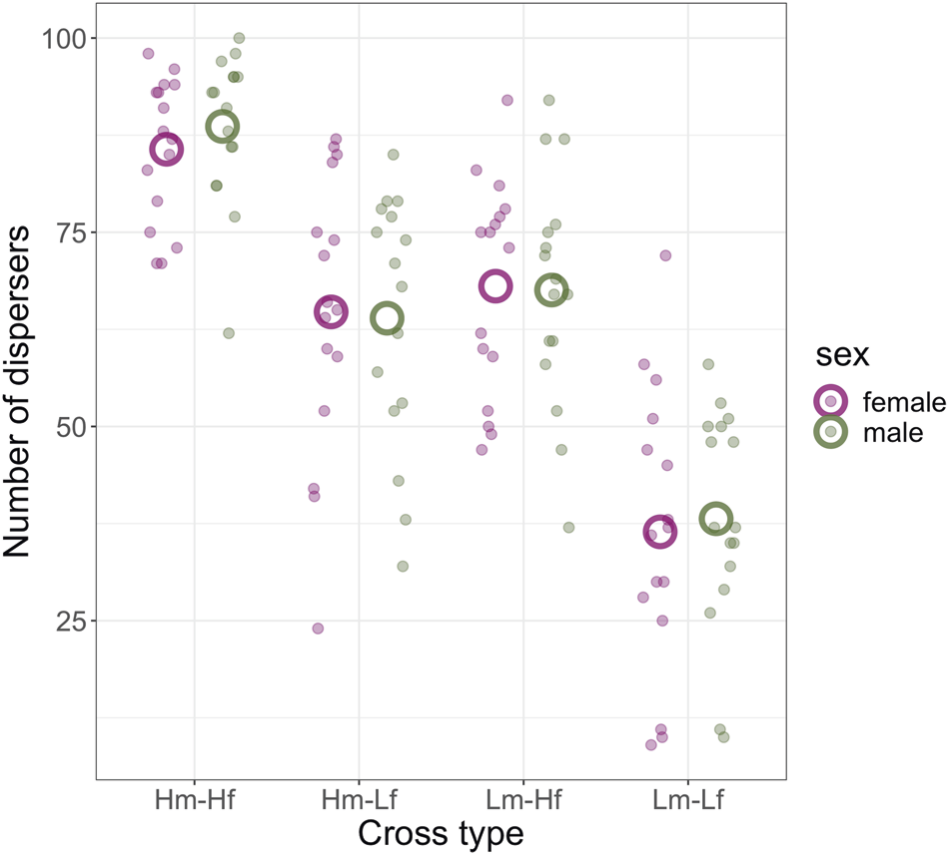
Number of dispersers from assayed mixed-sex populations of 200 *T. castaneum* offspring from crosses within and between dispersal selection regimes. On the X-axis, ‘H’ and ‘L’ represent the selection regime and ‘m’ and ‘f’ the sex of parents, e.g. Hm-Lf indicates that the male parent came from a high selection line and the female parent from a low selection line. Data for males and females are shown in purple and green respectively. Small filled points show experimental measures from individual crosses, while large hollow points show predictions of a GLMM fitting the effect of the interaction of sex and cross type on number of dispersers, while controlling for line ID.

When testing the individual fixed effects of whether cross type and sex predicted dispersal, we found a significant effect of cross type (Fig. 2; Table 2). Within-high regime crosses showed significantly higher dispersal than all other crosses, while dispersal from within-low regime crosses was significantly lower than all other cross types. Both reciprocal between regime crosses showed dispersal propensities intermediate to the within-regime crosses but did not different significantly from each other. No effect of sex on dispersal was found. No significant interaction was seen between cross type and sex (Table 2).

**Table 2:** Mixed models testing the dispersal behaviour of offspring of reciprocal crosses within and between *T. castaneum* lines experimentally selected for high and low dispersal propensity.

	Estimate	*SE*	*p*
(Intercept)	63.93	3.33	<0.001
Cross type [Hm-Lf]	(reference)		
Cross type [Hm-Hf]	22.81	2.47	<0.001
Cross type [Lm-Hf]	3.47	2.47	0.162
Cross type [Lm-Lf]	−21.16	2.47	<0.001
Sex	0.83	1.74	0.636
Cross type [Hm-Lf] x sex [female]	(reference)		
Cross type [Hm-Hf] x sex	3.75	4.98	0.453
Cross type [Lm-Hf] x sex	0.31	4.98	0.95
Cross type [Lm-Lf] x sex	2.5	4.98	0.62

Above the dashed line results are from a model fitting the individual effects of predictors, below the line results are from an equivalent model fitting the interaction between sex and cross type, with ‘Hm-Lf’ as the reference category. In both, we modelled the random intercept of line ID (model 1: Var = 116.26, SD = 10.78; model 2: Var = 116.26, SD = 10.78).

### Experimental results**—**dispersal phenotypes of inbred lines

**Fig. 3:**
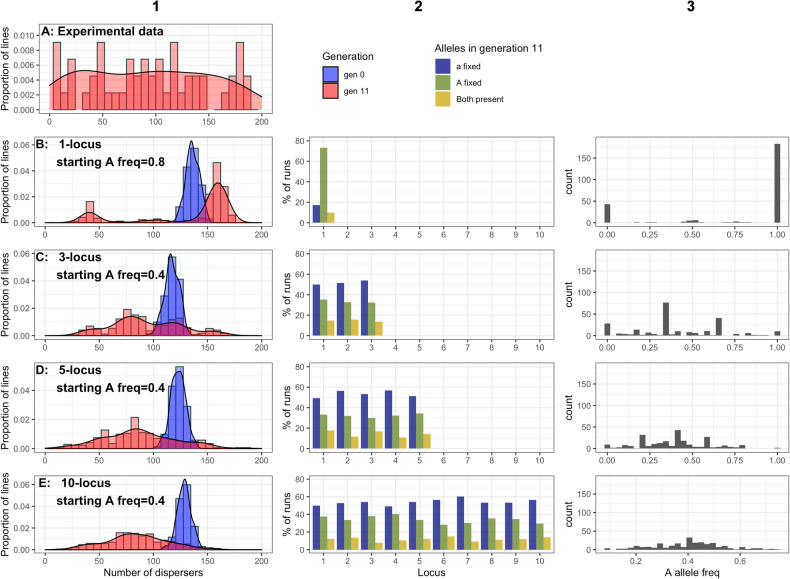
Combined data from experimental (column 1, row A) and simulated (all other panels) evolution experiments selecting on dispersal behaviour. Column 1: (**A**) Experimental results showing the number of dispersers from each of 64 lines of *T. castaneum* following 11 generations of extreme inbreeding and (**B**–**E**) data from agent-based simulations designed to model the same inbreeding design and dispersal assay as used experimentally. Numbers of dispersers are shown prior to (blue) and following (red) 11 generations of inbreeding. Sample sizes for simulations were 250 independent populations for each genetic architecture, each modelling the trait as controlled by either 1, 3, 5 or 10 additive biallelic loci (rows B-E respectively). For each simulated architecture, starting dispersal allele frequencies were 0.8, 0.4, 0.4 and 0.4 in these architectures respectively, this and other parameters were selected as those maximising agreement with experimental results during a parameter scan (Fig. 4). For simulated data, additional columns display: 2) Allele outcomes at each locus at generation 11, i.e. whether either allele had become fixed, or both alleles remained in the population. 3) The distribution of allele frequencies averaged across loci for each individual simulated line.

Inbred lines displayed a range of dispersal propensities, from 0–188/200 beetles dispersing (Fig. 3, row A), with a distribution not differing from unimodal (D = 0.04, *p* = 0.72).

### Simulated results**—**comparison of simulated to experimental selection on dispersal

**Fig. 4:**
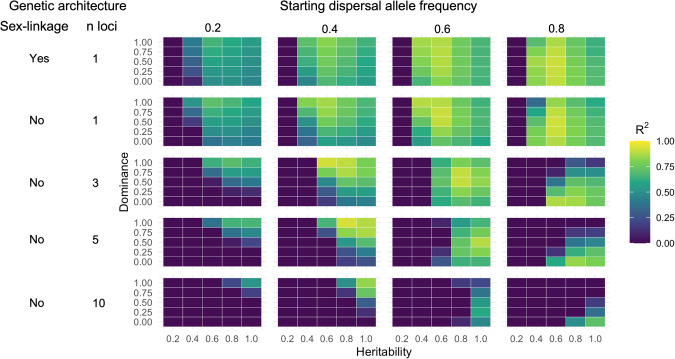
Outputs from parameter scans using an agent-based simulation designed to model an emigration selection experiment using *T. castaneum* under different trait architectures. Colours represent *R*^*2*^ values assessing the fit of a model defined on experimental data, and applied to simulated data across scenarios comprising combinations of dominance (d), trait heritability (h) and starting dispersal allele frequency (A), across a sex-linked single-locus, and unsex-linked single-, 3-, 5- and 10 locus architectures.

Considering only non-sex-linked architectures, which varied in the number of underlying loci, 20 of 400 scenarios had R^2^ values of > = 0.80, representing a good fit of the experimental model to the simulated data. Of these, 15 scenarios came from single-locus simulations, 11 from 3-locus, seven from 5-locus and one from a 10-locus simulation. Architectures varied in the parameter space over which they could simulate a good fit to the experimental data (Fig. 4), with less functional parameter space observed with increasing number of loci. Individual scenarios with good fit were observed across a wide range of parameter values.

Heritability values (h) required for good model fits were *h* ≥ 0.6 under single- and 3-locus architectures (Fig. 4). Good model fits generally required higher heritability values when dominance (d) was low, especially at higher numbers of controlling loci. Within each architecture, fits were poorest at low starting dispersal allele frequency (A = 0.2), though good fits were seen across the rest of the range of this parameter (*A* = 0.4, *A* = 0.8). Dominance (d) showed a strong negative interaction with starting dispersal allele frequency (A), with higher values of dominance required when dispersal allele frequency was low.

Considering the sex-linked single-locus model, the functional parameter space and parameter values of maximum fits were almost identical to the non-sex-linked version. Sex biases in dispersal were observed to be greater in sex-linked than non-sex-linked simulations under some parameter scenarios, though these differences were not of large magnitude. In high dispersal lines simulated with a sex-linked architecture, high dominance values (*d* = 1) resulted in greater dispersal in females than in males, with this pattern being strongest at low starting dispersal allele frequency and becoming minimal at the highest tested starting dispersal allele frequency (*A* = 0.8). In low selection lines, the inverse pattern was observed, with the lowest dominance values having male-biased dispersal, most pronounced at the maximum starting dispersal allele frequency and minimal at *A* = 0.2. In contrast, the non-sex-linked scenarios showed small stochastic variations across parameter space in both high and low selection lines.

Simulated crosses between selection lines generated divergent outcomes between sex-linked and non-sex-linked single-locus architectures (Table 3). Under both architectures, the offspring of within-high and within-low regime crosses showed high and low dispersal behaviour respectively, in both sexes. In contrast, behaviour of offspring of between regime crosses differed between architectures: as expected, both sexes showed intermediate behaviour under a non-sex-linked architecture, as did the female offspring under sex-linkage; but male offspring from this architecture inherited the behavioural phenotype of their mother’s regime. This pattern was reflected by the model, where a significant interaction was seen between cross type and sex under the sex-linked, but not non-sex-linked architecture (Table 3). Applying the mixed model fitting the interaction of cross type and sex, generated using experimental data, to the output from simulating each architecture resulted in better fit to the non-sex-linked data (R^2^ = 0.63) than to the sex-linked data (R^2^ = 0.50).

**Table 3:** General linear models of the dispersal behaviour of offspring of reciprocal crosses within and between simulated lines selected for high and low dispersal propensity, under sex-linked and unsex-linked genetic architectures.

	Sex-linked	Unsex-linked				
	Est	SE	*p*	Est	SE	*p*
(Intercept)	34.46	1.37	<0.001	51.04	0.69	<0.001
Cross type [HmLf]	(reference)					
Cross type [HmHf]	45.58	1.73	<0.001	28.33	0.87	<0.001
Cross type [LmHf]	30.26	1.73	<0.001	−0.66	0.87	0.447
Cross type [LmLf]	−14.94	1.73	<0.001	−30.19	0.87	<0.001
Sex	0.48	1.26	0.695	−0.15	0.61	0.807
Cross type [HmLf] x [female]	(reference)					
Cross type [HmHf] x male	29.32	1.71	<0.001	−2.62	1.73	0.13
Cross type [LmHf] x male	59.96	1.71	<0.001	1.8	1.73	0.298
Cross type [LmLf] x male	30.08	1.71	<0.001	0.54	1.73	0.755

Above the dashed line results are from a model fitting the individual effects of predictors, below the line results are from an equivalent model fitting the interaction between sex and cross type, with ‘HmLf’ as the reference category (model 1, sex-linked: Var = 116.26, SD = 10.78; model 2: Var = 116.26, SD = 10.78).

When simulating inbreeding with different numbers of loci, the patterns of alleles remaining in generation 11 were always similar across loci within each architecture. Patterns were also similar across architectures with the same starting allele frequency, with the frequency of an allele leading to its being fixed more often (Fig. 3, rows B-E, column 2).

### Simulated results**—**dispersal of simulated inbred lines

Within the simulated inbred lines the distribution of dispersal at generation 0 was unimodal with mean values of 100–150 dispersers under all architectures (Fig. 3; column 1). By generation 11 the distribution displays two obvious modes when modelled with a single-locus architecture. These observations were confirmed with dip tests for multimodality, indicating that the single-locus simulation showed a significantly non-unimodal distribution of numbers of dispersers under the parameter set that gave the greatest agreement with results of experimental selection (*D* = 0.06, *p* < 0.001). This remained true under all other single-locus scenarios which resulted in high agreement (*R*^2^ of >0.8; *D* < 0.14, *p* < 0.001). As expected, the same pattern was observed in allele frequencies; both the best performing (*D* = 0.09, *p* < 0.001) and all other high performing parameter sets (*R*^2^ > 0.8) showed significantly non-unimodal distributions (*D* < 0.18, *p* < 0.001).

Under the 3-, 5- and 10-locus architectures the distribution of dispersal outcomes in generation 11 was shifted to the left but retained an apparently unimodal distribution. However, many scenarios under the 3- and 5-locus architectures showed statistical multimodality, even after adjustment for multiple comparisons (3-locus: 0.03 < *D* < 0.09, 0.4<*p* < 0.001; 5-locus: 0.02 < *D* < 0.04, 0.02<*p* < 0.99). All scenarios under these architectures also showed multimodality of allele frequency distributions (*D* < 0.13, *p* < 0.001). When the trait was controlled by 10 loci, neither the number of dispersers (*D* = 0.02, *p* = 0.86), nor the underlying allele frequency showed significantly multimodal distributions (*D* = 0.03, *p* = 0.12). Across all architectures, peaks in disperser numbers appear to correspond to peaks in dispersal allele frequency (Fig. 3, columns C and D).

When single-locus simulation results were subsampled down to experimental sample size (*n* = 64) the bimodal pattern remained strong, though the pattern was no longer significantly different from unimodality (Hartigan’s dip test; *D* = 0.06, *p* = 0.07), but did show multimodal allele frequency distribution (*D* = 0.07, *p* < 0.05).

## Discussion

*Tribolium* beetles are significant agricultural pests and important model organisms, whose life history is characterised by bouts of dispersal and colonisation (Dawson 1977), yet the genetic basis of dispersal behaviour in this species is unresolved. Here, we demonstrate rapid evolution of dispersal behaviour under strong selection, mostly as a response to selection for low dispersal but also as a significant increase in dispersal in high selection lines. We find no evidence of sex biases in dispersal in offspring of crosses, suggesting that the trait is not sex-linked. Simulations show that feasible parameter space over which simulated data can approximate experimental data exists under scenarios where the dispersal trait is controlled by one or few loci, but not many loci. However, levels of dispersal in experimentally inbred lines as compared with simulations, indicate that a single locus model is not well supported. Considered together, we suggest that these findings support an oligogenic architecture underlying dispersal in *Tribolium castaneum*.

Past work on *Tribolium* has provided evidence of a genetic basis to dispersal behaviour, which our results corroborate (Schurr and Bolduan 1967; Ogden 1970a, 1970b; Ritte and Lavie 1977; Korona 1991; Ruckman and Blackmon 2020). We saw a rapid response to selection on dispersal, creating extreme divergence between the behaviour of high and low dispersal selection regimes over just two generations in our *T. castaneum* lines. Such a result clearly demonstrates that variation for the trait was present in our stock *T. castaneum* population, despite more than ten years in the laboratory. This raises the question of how variation for dispersal behaviour is maintained in laboratory populations with no ability to disperse, especially since dispersal appears to trade-off with fecundity (Zirkle et al. 1988). It may be that positive correlates of dispersal observed in this species (e.g. shorter development time; Zirkle et al. 1988) are sufficient to keep dispersal phenotypes common. Interestingly, a greater reduction was observed in dispersal in low selection lines than the increase seen in high dispersal lines, indicating that dispersal tendency was already high in the original population. This is in contrast to previous studies which have shown lower initial rates and larger increases in dispersal in high selection lines (Ritte and Lavie 1977; Ruckman and Blackmon 2020). One explanation for this may be differences in methodology; our approach, with assays conducted on mixed-sex groups having had prior opportunity to mate, is more ecologically realistic than methods measuring dispersal in sibling groups (Ritte and Lavie 1977) and single-sex groups of virgins (Ruckman and Blackmon 2020), where movement may be driven by mate-searching. This observation may be explained by differences in the lines used; our KSS populations, having been bred to combine global *T. castaneum* diversity, are likely diverse relative to other strains and perhaps better capture the high level of dispersal present in wild populations existing in patches of ephemeral habitat, even though the strains used in both other studies were recently collected from the wild. Results are also consistent with differences in husbandry practices between laboratories (personal comm. Blackmon), our laboratory may have inadvertently selected for increased dispersal in stock populations prior to the study, perhaps by retrieving breeding individuals from the surface of the fodder to parent each subsequent generation. If this is the case, it suggests a relationship between dispersal and use of different strata of the fodder, which may warrant further investigation.

Our results, comparing experimental selection to selection simulated over a range of genetic parameters and architectures, broadly support an oligogenic architecture of dispersal. Models involving one, three and five, and to a lesser degree 10, additive loci were capable of agreeing with our empirical results under at least one combination of heritability, dominance and starting dispersal allele frequency. Therefore our findings suggest that if additive variance predominates in the genetic determination of dispersal, few loci are major contributors to the trait. However, we find no evidence that there needs to be only a single locus to account for the observed rapid change in behaviour under selection. Indeed, results from simulations and experiments using inbred lines provide tentative evidence that the trait involves more loci. Theoretically, under architectures with fewer interacting loci, fixation of alleles resulting from inbreeding has a larger impact on the focal trait. We observed this in simulations, where under a single locus the fixation of dispersal alleles was detectable as multimodality in the dispersal distribution. Importantly, the experimental results did not show multimodality in their dispersal distribution, suggesting that the trait may involve more than one locus. This finding is counter to the conclusions of Ritte and Lavie (1977), who postulated that *Tribolium* dispersal was controlled by a single locus. Recent theoretical work suggests that rapid adaptation can also occur with complex genetic architectures, including highly polygenic ones (Jain and Stephan 2017a, 2017b), and that the evolution of dispersal may be more rapid under the control of larger number of loci (Weiss-Lehman and Shaw 2022); counter to the prevailing wisdom that selective sweeps on large-effect loci are the predominant mode of rapid responses to selection (Messer and Petrov 2013). Such rapid evolution of dispersal can be important, for example, during range expansion; there is theoretical evidence that evolving increased dispersal at the range front can prevent the accumulation of expansion load (Peischl and Gilbert 2020). As a species that relies on ephemeral habitat (Dawson 1977), and has undergone extensive natural and human-mediated range expansion, this process may well have been important in the evolutionary history of *T. castaneum*. Oligogenic architectures of dispersal traits have been found in other insect taxa, notably in the Glanville Fritillary butterfly (*Melitaea cinxia*), where an epistatic interaction between two genes influences metapopulation dynamics through variation in dispersal (Hanski 2011; Niitepõld and Saastamoinen 2017).

We found that offspring of crosses between high and low dispersal lines demonstrated intermediate dispersal behaviour. This is consistent with some prior work (Ritte and Lavie 1977), but contrasts with one study which found that the dispersal of between treatment crosses was higher than in their high dispersal lines (Ruckman and Blackmon 2020). This elevated dispersal behaviour of cross offspring contributed heavily to their conclusion that epistatic variance outweighs additive variance in dispersal. This difference could, again, be due to their use of virgin single-sex groups, as opposed to mixed-sex groups, in dispersal assays, though further experiments would be needed to determine this. We were able to approximate through simulation both the response to selection and behaviour of the offspring of crosses observed in experiments, using only simple additive interactions between loci, without invoking epistasis. This is not to say that epistasis could not also account for the observed data, which we did not test, but that our observations are consistent with a simple additive architecture.

When we crossed experimental lines selected for high and low dispersal, offspring displayed the pattern expected under a non-sex-linked genetic architecture. Further, we included a single-locus sex-linked architecture among those which we simulated selection on dispersal. Experimental results agreed more closely with simulated crosses performed under an assumption of non-sex-linkage than with sex-linkage. These findings suggest that the dispersal trait on which we have selected is not controlled by a single sex-linked locus, as posited by Ritte and Lavie (1977).

Sex-biased dispersal is common in nature (Trochet et al. 2016), and sex-linkage of dispersal genes is likely important to its evolution (Brom et al. 2018). In our simulations, we saw sex-biased dispersal in only a small subset of scenarios of single loci sex-linked architecture. Complete dominance of either allele resulted in sex-biased dispersal, with females dispersing more when the dispersal allele was dominant and males dispersing more when the non-dispersal allele was dominant, as expected in an XX/XY system. When dominant alleles are rare, the strength of this effect is increased, explaining the interaction we observed between sex-biased dispersal and starting allele frequency in these simulations. Experimentally, while we saw no sex bias in the dispersal of high selection lines, we saw evidence of a weak female bias in low selection lines**—**a pattern not seen in our simulations, suggesting a cause not captured by our model. Similar results have been obtained by other studies of mixed-sex populations (Ogden 1970b; Ziegler 1977), suggesting that the male-biased dispersal seen in groups of siblings (Ritte and Lavie 1977) or single-sex virgins (Prus 1963; Ruckman and Blackmon 2020), is the result of either the mating-status, population sex ratio or relatedness. All of these are known to be important mediators of individual dispersal in other systems (Clobert et al. 2012), and in the case of relatedness, in *Tribolium* larvae (Jasieński et al. 1988). In particular mating status has recently been shown to alter dispersal decisions in the northern tamarisk beetle, *Diorhabda carinulata* (Clark et al. 2022). The contrast between our results and those using virgin or mated single-sex groups of *Tribolium* (Ritte and Lavie 1977; Ruckman and Blackmon 2020) highlights the importance of mating status and/or social environment in this system, and the ability of individuals to perceive and alter behaviour in response. Social environment is known to modify developmental life-history traits in *Tribolium* (Ellen et al. 2016), however, tests of mating status on flight have shown mixed results (Perez-Mendoza et al. 2011).

Our results support the view that dispersal in *Tribolium* is under the control of a small number of loci. A logical next step would be to investigate where these genes are and how they act to determine the phenotype. That *Tribolium* dispersal appears to be oligogenic (rather than polygenic) means that a genome-wide association approach may be able to detect loci across lines selected for differential dispersal. Known genes with large effects on dispersal in insects commonly have broad physiological, metabolic or neurological functions and are important beyond their effects on movement (Goossens et al. 2020); and it will be interesting to discover how genes are acting to control dispersal in *Tribolium*. It would also be instructive to explore whether dispersers and non-dispersers differ in measures such as leg length, activity and movement pattern. For example, a difference in dispersal but not leg length or movement pattern might suggest a physiological rather than a morphological or neurological basis. Such findings could then be thought of in relation to any candidate genes identified using genomics. *Tribolium* is a leading model system for RNA interference (Klingler and Bucher 2022), and candidate dispersal genes would be attractive targets for knockouts which, if effective, could present a valuable method of controlling this prolific pest.

Dispersal is an important aspect of the life history of many species, and its study in taxa such as pests and invaders could bring significant agroeconomic and biosecurity benefits. However, as a complex behaviour is challenging to study. We provide evidence that dispersal in the pest insect *T. castaneum* is under oligogenic control, opening up the possibility of identifying the loci involved using molecular genomics.

### Supplementary information

Supplementary material

## Data Availability

All data files, simulation scripts and analysis scripts associated with this article are available in Dryad (10.5061/dryad.c866t1gcb).
