# Supplementary material for: Genetic architecture of dispersal behaviour in the post-harvest pest and model organism Tribolium castaneum
Source: Heredity (Edinb). 2023 Jul 29;131(4):253–62. doi: 10.1038/s41437-023-00641-6 (PMC10539327; doi:10.1038/s41437-023-00641-6)
Supplement: Supplementary file 1 — Supplementary material [file 41437_2023_641_MOESM1_ESM.pdf]

Supplementary material

Simulation methods

We simulated starting populations of 200 individuals, equal to that of experimental assays, and assigned genotypes probabilistically using specified starting allele frequencies (in runs using a sex-linked architecture the probability of receiving a Y as the second allele was equal to the sex ratio). Individuals were each assigned binary dispersal outcomes for each dispersal opportunity of the simulated assay, according to their genotype. The following equations were used to calculate the contribution of a single locus to the probability of dispersing given specified values of dispersal trait heritability (h) and dominance (d), which were then used to draw outcomes from a Bernoulli distribution. For an aa genotype this was:

(1) $$\frac{1-h}{2}$$

for an Aa genotype;

(2) $$\frac{2hd-h+1}{2}$$

And for an AA genotype;

(3) $$\frac{h+1}{2}$$

Figure S3 shows how the probability of dispersal varied for each genotype across the possible combinations of h and d.

Following the format of the dispersal selection experiment, in the model 30 individuals were chosen at random from those that emigrated zero or three times to act as parents of the subsequent generation of low and high selection regime lines respectively. From this point, the model simulated and tracked low and high lines as discrete populations. Within each selection regime, each parental female was mated with between one and three males, with equal probability. The number of offspring produced by each female was chosen from a normal distribution with a mean of 100 and standard deviation of 25 (approximating typical values for *T.castaneum,* eg. (Vasudeva *et al.*, 2021). At each simulated locus independently, each of these offspring was assigned its first allele randomly chosen from the female parent and its second allele from one of that female's mates with equal probability. These new individuals were then assigned dispersal outcomes as above, and the cycle of reproduction and dispersal assays repeated over a total of 5 generations.

Comparison of 3-opportunity and 1-opportunity dispersal assays

To ensure the continuity of inferences between 1- and 3-opportunity dispersal assays, we used dispersal data from generation 5 and fitted the same model to both 1- and 3-opportunity data and compared the results. First a model was fitted to the full 3-opportunity dataset, then the same model was separately fitted to the same dataset filtered to just the first of three dispersal opportunities, to represent a 1-opportunity assay. Initially we applied the same GLMM to the 1-opportunity data as has been described in the main analysis, however with the lower amount of data these models had singular fit (the variance explained by the random effect was zero). We

therefore simplified the effects structure by dropping the random effect of selection line and fitted a GLM to both datasets - incorporating the response variable, with selection regime and block ID as fixed effects.

Data from both 1- and 3-opportunity dispersal assays were capable of showing that individuals from the high emigration selection regime were significantly more likely to emigrate than low regime beetles. The greater resolution of data over three dispersal opportunities led to a larger effect of selection regime using these data (GLM, $\beta=-1.58$, SE=0.06, $p<0.001$) relative to the data over a single dispersal opportunity (GLM, $\beta=-0.62$, SE=0.02, $p<0.001$).

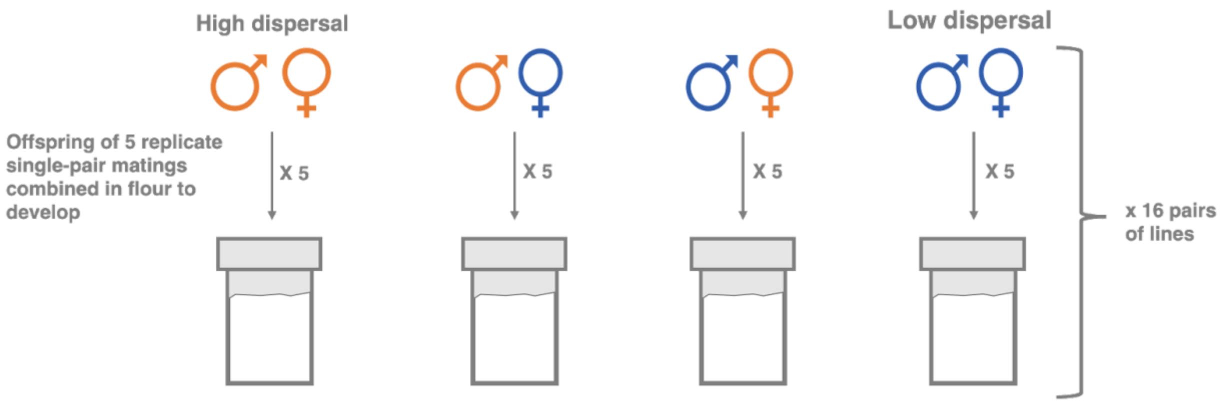

Figure S1. Representation of the method used to obtain offspring for dispersal assays of reciprocal crosses between pairs of high- and low-dispersal selection lines of *T.castaneum*. For each of 16 pairs of lines, each of 4 cross types was replicated with 5 single-pair matings, with egg-containing fodder combined to develop as a population.

Table S1. The genetic architectures simulated, and the range and increments used for each of the parameters heritability (h), dominance (d) and starting allele frequency (A). One combination of these parameters is termed a 'scenario'. The model was run using all 100 unique scenarios for each of the 5 genetic architectures tested.

Genetic architecture		Simulated scenarios: a unique combination of h, d and A		
		Range [increment] of simulated parameters		
Number of loci	Sex-linked	Heritability (h)	Dominance (d)	Starting allele frequency (A)
1	Yes	0.2 - 1 [0.2]	0.2 - 1 [0.2]	0.2 - 0.8 [0.2]
1	No	0.2 - 1 [0.2]	0.2 - 1 [0.2]	0.2 - 0.8 [0.2]
3	No	0.2 - 1 [0.2]	0.2 - 1 [0.2]	0.2 - 0.8 [0.2]
5	No	0.2 - 1 [0.2]	0.2 - 1 [0.2]	0.2 - 0.8 [0.2]
10	No	0.2 - 1 [0.2]	0.2 - 1 [0.2]	0.2 - 0.8 [0.2]

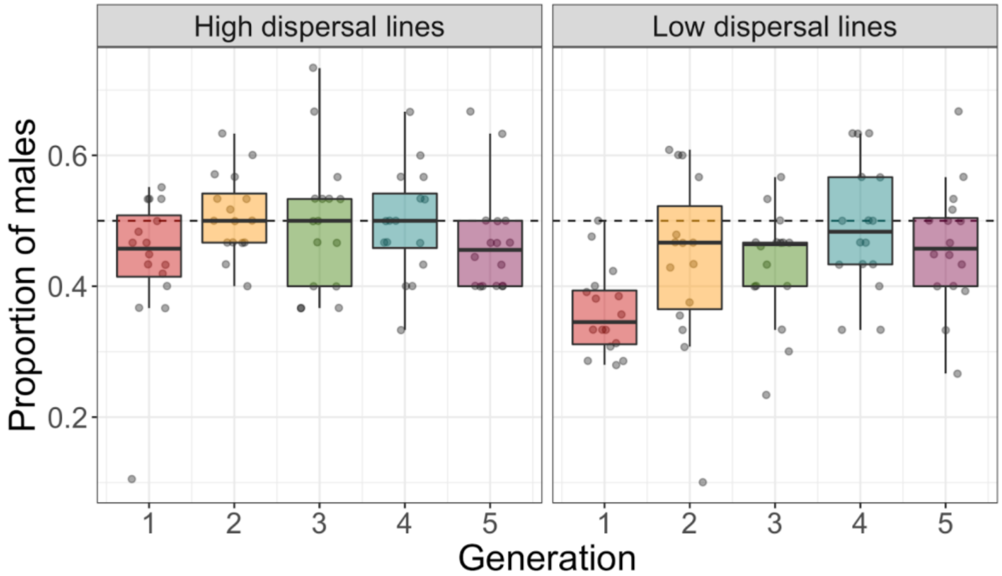

Figure S2. Sex ratio within groups of 30. *Tribolium castaneum* beetles selected to parent the next generation of high and low dispersal lines during an artificial selection experiment. The dashed line represents an equal sex ratio.

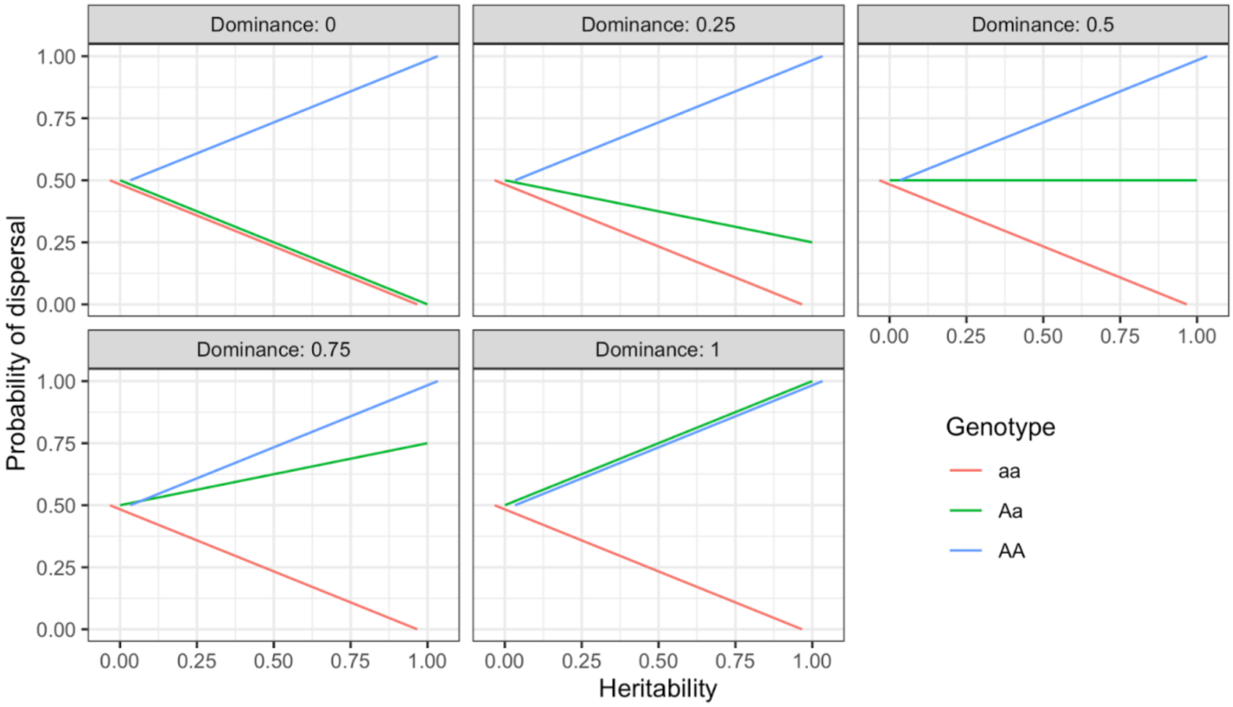

Figure S3. Dispersal probabilities of simulated individuals having each possible single-locus biallelic genotype, at combinations of heritability, and dominance (panels). Where lines occupy the same plot space they have been shifted so that both are visible, i.e. blue and red lines show reflective symmetry across the line y=0.5.
